# Landscape of immune infiltration in entorhinal cortex of patients with Alzheimerʼs disease

**DOI:** 10.3389/fphar.2022.941656

**Published:** 2022-09-28

**Authors:** Hui Zhang, Silu Cao, Yaru Xu, Xiaoru Sun, Miaomiao Fei, Qi Jing, Xiaodong Xu, Jinxuan Tang, Bing Niu, Cheng Li

**Affiliations:** ^1^ Department of Anesthesiology and Perioperative Medicine, Shanghai Fourth People’s Hospital, School of Medicine, Tongji University, Shanghai, China; ^2^ Shanghai Key Laboratory of Anesthesiology and Brain Functional Modulation, Shanghai, China; ^3^ Translational Research Institute of Brain and Brain-like Intelligence, Shanghai Fourth People’s Hospital, School of Medicine, Tongji University, Shanghai, China; ^4^ Clinical Research Center for Anesthesiology and Perioperative Medicine, Tongji University, Shanghai, China; ^5^ Department of Anesthesiology, Shanghai Tenth People’s Hospital, School of Medicine, Tongji University, Shanghai, China; ^6^ School of Life Sciences, Shanghai University, Shanghai, China

**Keywords:** Alzheimer’s disease, entorhinal cortex, immune, transcriptomic, drug

## Abstract

Alzheimer’s disease (AD) is one of the most common neurodegenerative diseases and manifests as progressive memory loss and cognitive dysfunction. Neuroinflammation plays an important role in the development of Alzheimer’s disease and anti-inflammatory drugs reduce the risk of the disease. However, the immune microenvironment in the brains of patients with Alzheimer’s disease remains unclear, and the mechanisms by which anti-inflammatory drugs improve Alzheimer’s disease have not been clearly elucidated. This study aimed to provide an overview of the immune cell composition in the entorhinal cortex of patients with Alzheimer’s disease based on the transcriptomes and signature genes of different immune cells and to explore potential therapeutic targets based on the relevance of drug targets. Transcriptomics data from the entorhinal cortex tissue, derived from GSE118553, were used to support our study. We compared the immune-related differentially expressed genes (irDEGs) between patients and controls by using the limma R package. The difference in immune cell composition between patients and controls was detected *via* the xCell algorithm based on the marker genes in immune cells. The correlation between marker genes and immune cells and the interaction between genes and drug targets were evaluated to explore potential therapeutic target genes and drugs. There were 81 irDEGs between patients and controls that participated in several immune-related pathways. xCell analysis showed that most lymphocyte scores decreased in Alzheimer’s disease, including CD4^+^ Tc, CD4^+^ Te, Th1, natural killer (NK), natural killer T (NKT), pro-B cells, eosinophils, and regulatory T cells, except for Th2 cells. In contrast, most myeloid cell scores increased in patients, except in dendritic cells. They included basophils, mast cells, plasma cells, and macrophages. Correlation analysis suggested that 37 genes were associated with these cells involved in innate immunity, of which eight genes were drug targets. Taken together, these results delineate the profile of the immune components of the entorhinal cortex in Alzheimer’s diseases, providing a new perspective on the development and treatment of Alzheimer’s disease.

## Introduction

Alzheimer’s disease (AD) is one of the most common neurodegenerative diseases (Jack et al., 2010). Approximately 50 million people worldwide suffer from dementia, and by 2050, more than 100 million people will experience dementia, which places a heavy economic burden on societies and families (Lane et al., 2018; Jing et al., 2021). The clinical features of AD include progressive memory loss and cognitive dysfunction. The main pathological changes in AD include β-amyloid deposition, neurofibrillary tangles, neuronal loss, synaptic dysfunction, and neuroinflammation in the brain (Serrano-Pozo et al., 2011; Jack et al., 2013; Spires-Jones & Hyman, 2014; Oliveros et al., 2022; Peng et al., 2022). Despite significant advancements in assessing AD, both from basic and clinical studies, there is currently no effective treatment to prevent or reverse AD. Therefore, the etiology and pathogenesis of the disease require further study and elucidation. Moreover, there is an urgent need for effective drugs to prevent and delay the progression of AD. In recent years, increasing research evidence has revealed that neuroinflammation plays a crucial role in AD (Heneka et al., 2015; Ransohoff, 2016; Lindestam Arlehamn et al., 2019). Central microglia, astrocytes, and peripheral monocytes are considered to be the main cells involved in neuroinflammation. Microglia can penetrate cell surface receptors and is hypothesized to play an important role in the inflammatory response in AD (Paresce et al., 1996; Bamberger et al., 2003; Liu et al., 2005; Stewart et al., 2010). The results from animal studies have suggested that peripheral mononuclear cell infiltration is associated with amyloid plaques (Simard et al., 2006). Furthermore, a mouse model demonstrated that peripheral mononuclear phagocytes play a critical role in reducing Aβ plaque accumulation (Simard et al., 2006). Pathological responses of astrocytes include reactive astrogliosis, a complex multistage pathologically specific response of astrocytes, which is usually considered to protect nerves and restore damaged nerve tissues (Sofroniew, 2009; Sofroniew & Vinters, 2010). Except for activated microglia, hypertrophic reactive astrocytes that accumulate around senile plaques are often observed both in postmortem human tissue from ADs (Medeiros & LaFerla, 2013) and in animal models with the disorder (Olabarria et al., 2010).

In addition to resident immune cells such as microglia and astrocytes, there are also infiltrating immune cells in the brain. Most infiltrating immune cells are mainly present in the border regions of the brain, and immune infiltrating cells are absent in the brain parenchyma under normal conditions (Cugurra et al., 2021). These border region cells can affect the brain by secreting cytokines, modulating adjacent epithelial and ependymal cells, and altering cerebrospinal fluid composition. These cells are involved in tissue homeostasis and may enter the brain parenchyma when an abnormality occurs; therefore, they may play a central role in promoting recovery and may also accelerate the pathological process. Many previous studies have concluded that inflammation has a damaging effect on neurons in the brains of patients with AD and the usage of anti-inflammatory drugs can reduce the risk of the disease (Aisen, 2002). In the past decade, there have been several reports of anti-inflammatory drugs, especially nonsteroidal anti-inflammatory drugs (NSAIDs), for the treatment of AD. Multiple meta-analyses have produced strong, generally consistent statistical evidence that the use of NSAIDs has resulted in a halved or even lower risk of developing AD (McGeer et al., 1996; Anthony et al., 2000). Therefore, exploring the immunological differences between patients with AD and controls may provide evidence for the treatment of AD.

The entorhinal cortex is a vital link between the cerebral cortex and hippocampus, and it plays a crucial role in the formation and retrieval of memory (de Calignon et al., 2012). The molecular mechanism of entorhinal cortex alterations is significant for the prevention and treatment of AD. Our study aimed to provide a landscape of different immune cell compositions in the entorhinal cortex between patients with AD and controls based on the transcriptomics and signature genes of different immune cells by using the xCell algorithm (Aran et al., 2017). Combined with correlation analysis, genes related to immune microenvironment differences and the potential therapeutic targets involved in therapy were further identified. These results provide evidence to comprehensively understand the association between immune infiltration and disease in the brain parenchyma of patients with AD and to obtain new ideas for its prevention and treatment.

## Materials and methods

### Data collection

The Gene Expression Omnibus (GEO) database is an international public repository that archives and freely distributes high-throughput gene expression and other functional genomics datasets (Clough & Barrett, 2016). It mainly refers to gene sequencing data, including microarray, second-generation sequencing, and third-generation sequencing data, which can be downloaded by using the GEOquery package in the R programming environment (Davis & Meltzer, 2007). The GSE118553 dataset contain transcriptomic data of the entorhinal cortex, temporal cortex, frontal cortex, and cerebellum brain region from controls, asymptomatic AD, and AD subjects. Neuropathological evaluation for neurodegenerative diseases was performed in accordance with standard criteria (Patel et al., 2019). Among them, transcriptomic data from the entorhinal cortex of controls and ADs were extracted to support this study. The characteristics of the entorhinal cortex tissue from ADs and control samples in the GSE118553 dataset are shown in Table 1. According to the data processing instructions, the expression matrix was maximum likelihood estimation background corrected by using the R package MBCB (Allen et al., 2009), log2 transformed, and robust spline normalization by using the R package Lumi (Du et al., 2008). The data were annotated by using the GPL10558 platform. For multiple probes corresponding to the same gene, the average value of all probes was used as the gene expression value. The characteristics, such as age and sex, were also obtained from the GSE118553 dataset. We turned age into a categorical variable based on the median of it to facilitate comparison of baseline differences between ADs and controls. The differences between the two groups were studied using Chi-square tests in R. Propensity score matching (PSM), performed with the matching package, was applied to eliminate baseline differences.

**TABLE 1:** Characteristics of the entorhinal cortex tissue in AD and control samples in the GSE118553 dataset.

	Controls	ADs	*p*
Age (=<80/> 80)	17/7	13/24	0.006
Sex (Male/Female)	12/12	14/23	0.348

### Immune-related differentially expressed genes between Alzheimer’s diseases and controls

Differentially expressed genes (DEGs) between 37 ADs and 24 controls were detected with the limma package based on the cutoff criteria of a |log 2-fold change (FC)| > 0.5 and adjusted *p* value <0.05 (Ritchie et al., 2015). The immune-related gene (IRG) list was downloaded from ImmPort (https://www.immport.org/home) (Bhattacharya et al., 2018). Immune-related DEGs (irDEGs) were visualized using the Venn diagrams package (Nagpal et al., 2021), and the function of irDEGs was annotated by using Metascape (metascape.org), which is an online bioinformatic pipeline for multiple gene lists that allows effective gene function annotation and data-driven gene prioritization decisions (Zhou et al., 2019).

### Immune cells in samples

xCell, using a set of 10,808 genes for calculating the score of 64 immune and stromal cell types based on a novel gene signature–based method, was used to calculate the scores for immune cell infiltration in the entorhinal cortex tissue of each sample (Aran et al., 2017). The gene markers of each cell type are displayed in Supplementary Table S1 (Aran et al., 2017). According to the cell gene markers, a total of 34 immune cell types can be scored with xCell. A total of 21 of all immune cell types were lymphoid cells. The different cell type scores between ADs and controls were estimated with the Mann–Whitney *U* test, and a *p* value <0.05 was considered statistically significant. t-Distributed stochastic neighbor embedding (tSNE) analyses were performed with all cell signature genes to visualize all samples in 2D maps by using the tSNE algorithm (Kobak & Berens, 2019). Cell signature differentially expressed genes (csDEGs) were selected and displayed with a percent stacked bar chart.

### Correlation between cell signature genes and immune cells

To explore the gene potential causes of the differences in immune cells between ADs and controls, Pearson correlation analysis was used to assess the relationship between differentially expressed signature genes and corresponding cell scores, with a *p* value <0.05 considered statistically significant. The genes may play critical roles in causing cell differences between ADs and control. Similarly, the Pearson correlation analysis was performed to reveal the correlation between myeloid cells and lymphocytes in ADs, as well as the correlation between different cell types. Correlations between genes and each cell type were performed to detect potential genes that contribute to the association between cell types.

### Drug-targeted immune gene identification and functional annotation

In this study, we used three properties to identify potential therapeutic target immune genes. First, the gene is an immune cell signature gene or associated with immune cells, which has been obtained in the aforementioned analysis; second, the gene is associated with AD, which can be determined with GeneCards inferred functionality scores higher than 40 by the GeneCards database (https://www.genecards.org/); last, the gene is the target of drug action, which can be determined by the PharmGKB database (Hewett et al., 2002). The functions of target genes were annotated by Kyoto Encyclopedia of Genes and Genomes (KEGG) functional enrichment analysis. The expression of drug-targeted immune genes between the two groups is shown as a boxplot with ggplot2. Receiver operating characteristic (ROC) curves and areas under the ROC curve (AUCs) were examined by the pROC package to determine the predictive value of drug-targeted immune genes (Mandrekar, 2010). The relationship between potential targets and drugs was visualized by using Cytoscape 3.5.1 (Shannon et al., 2003).

### Prediction of candidate miRNAs

MicroRNAs (miRNAs) are small noncoding RNAs that direct posttranscriptional repression of many mRNAs and thereby regulate -diverse biological processes from cell proliferation and apoptosis to organ development and immunity (Bartel, 2009). We selected candidate miRNAs whose expression levels were correlated with those potential therapeutic target immune genes by using the ENCORI database (https://starbase.sysu.edu.cn/). The miRNAs with clipExpNum higher than t times and that regulated more than two target immune genes were screened, and they were considered to play critical value in participating in the expression level of selected drug-targeted immune genes. The expression of predicted miRNAs was obtained from GSE48552, which includes early-stage and late-stage AD subjects. The DESeq2 package was applied to detect differentially expressed miRNAs at different stages and between females and males.

## Results

### irDEGs between patients with Alzheimer’s disease and controls

**FIGURE 1:**
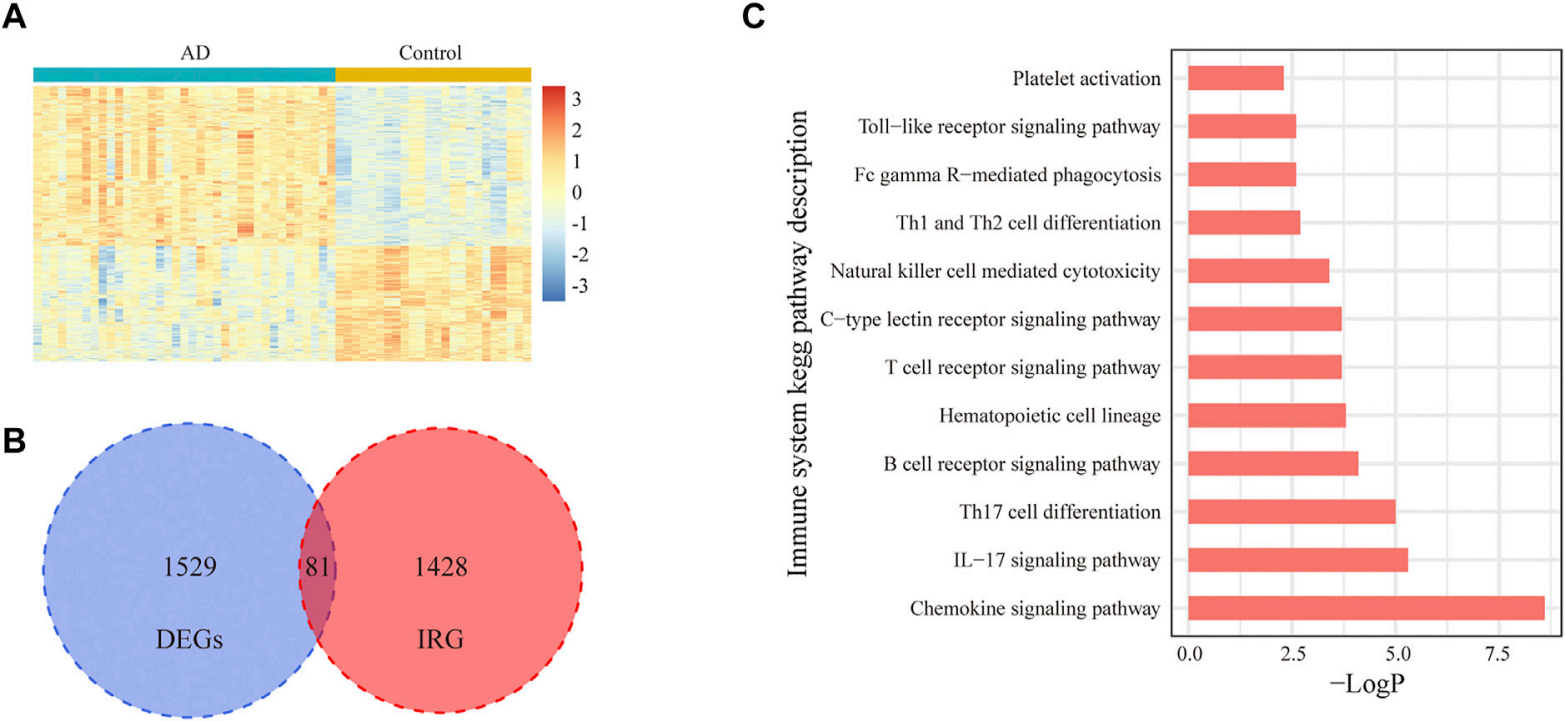
Expression of IRGs in the entorhinal cortex for AD and control samples in the GSE118553 dataset. **(A)**. Heatmap of 1,610 DEGs in the entorhinal cortex for AD and control samples in the GSE118553 dataset. The DEGs were filtered with |log2 fold change (FC)| > 0.5 and adjusted *p* value <0.05. **(B)**. In total, 81 IRGs in DEGs are displayed with a Venn diagram. **(C)**. In total, 12 immune system pathways were significantly enriched with 81 irDEGs.

In total, 31,426 mRNA expression profile data from 24 controls and 37 patients with AD in the GSE118553 dataset were used for this study. The characteristics of all samples are displayed in Table 1. There was a significant difference in age between the two groups (*p* = 0.006), whereas no significant difference was found in sex. The normalized mRNA expression levels in all samples are shown in Supplementary Figure S1. According to the screening criteria, 1,610 DEGs were detected between patients and controls. Among these genes, 81 DEGs were related to immunity. A heatmap of the differentially expressed genes and a Venn diagram of the irDEGs are shown in Figures 1A,B. A KEGG functional enrichment analysis of irDEGs in Metascape showed that these genes were significantly enriched in several immune system KEGG pathways, such as the chemokine signaling pathway, interleukin (IL)-17 signaling pathway, Th17 cell differentiation, B-cell receptor signaling pathway, hematopoietic cell lineage, T-cell receptor signaling pathway, C-type lectin receptor signaling pathway, natural killer cell–mediated cytotoxicity, Th1 and Th2 cell differentiation, Fc gamma R–mediated phagocytosis, Toll-like receptor signaling pathway, and platelet activation (Figure 1C). These results illustrate the involvement of immunity in AD progression at the transcriptome level.

### Differences in immune cells between patients with Alzheimer’s disease and controls

**FIGURE 2:**
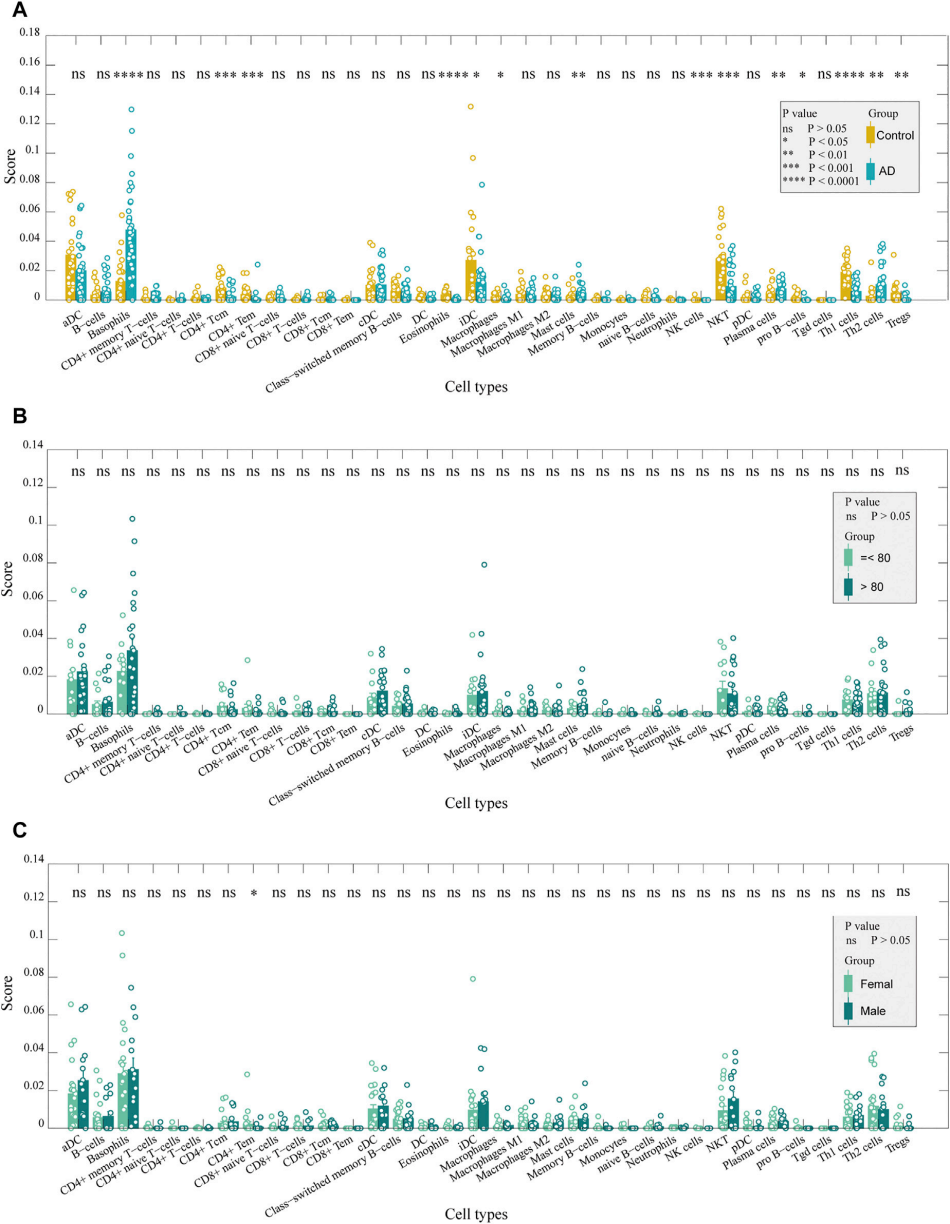
Immune cells in the entorhinal cortex between AD and control samples. **(A)**. Comparison of the scores of immune cells between AD and control samples: CD4^+^ Tc, CD4^+^ Te, Th1, NK, NKT, and pro-B cells, eosinophils, and Tregs were decreased in ADs (*p* < 0.05). In contrast, basophils, mast cells, plasma cells, and macrophages had elevated scores (*p* < 0.05). **(B)**. Comparison of immune cell scores between older and younger patients in AD samples: no significant difference was detected. **(C)**. Comparison of the score of immune cells between females and males in AD samples: only CD4^+^ Tem was significantly different between the two groups (*p* < 0.05).

**FIGURE 3:**
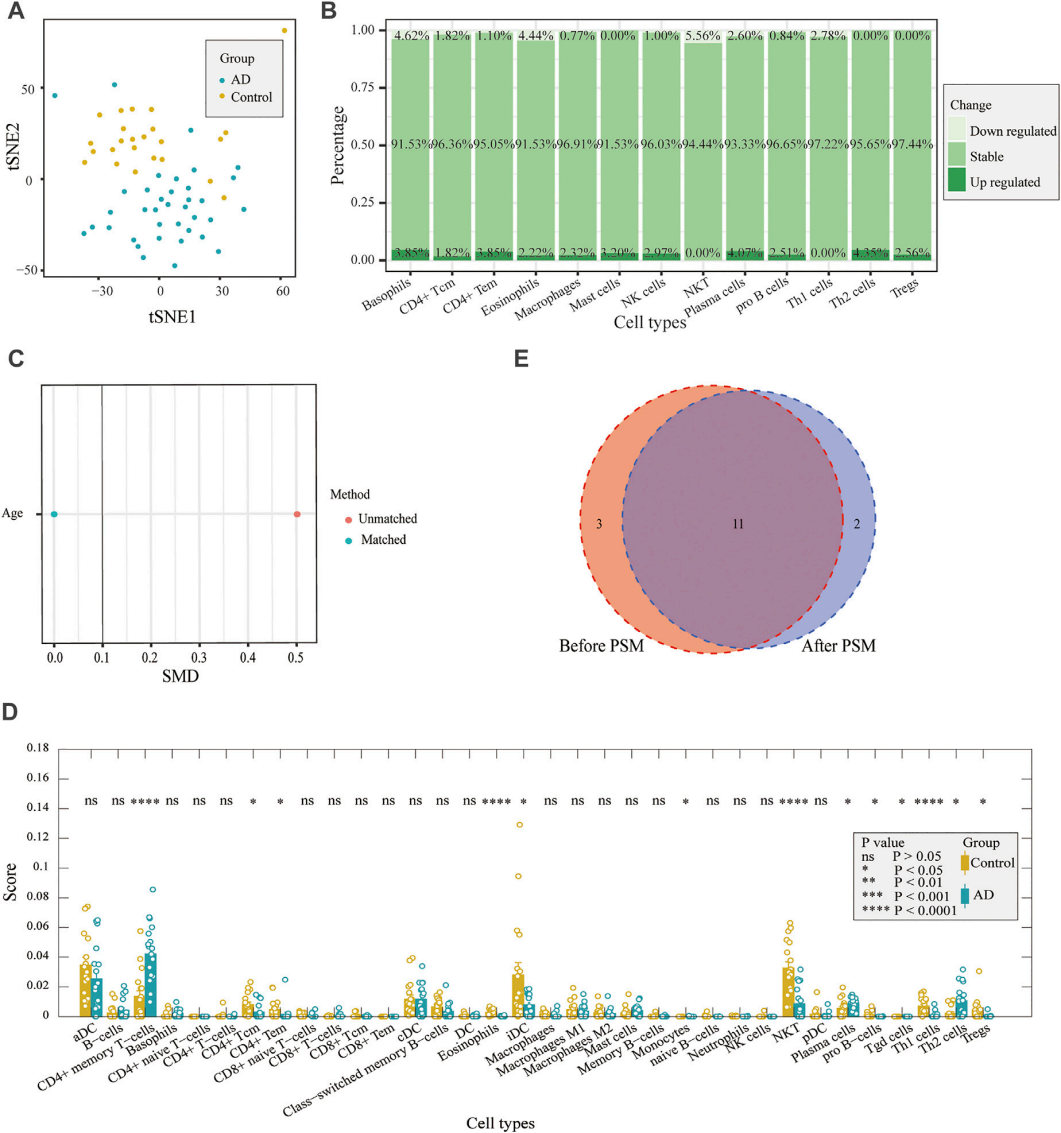
Correlation between differentially expressed marker genes and corresponding immune cells. **(A)** tSNE plot for AD and control individuals by using immune cell markers. **(B)**. DEG proportions in immune cell markers. **(C)** Difference in age was reduced after PSM. **(D)** Comparison of immune cell scores between AD and control samples after PSM. **(E)** Results both before PSM and after PSM are displayed with a Venn diagram. After PSM, changes in 11 immune cell scores were the same as before PSM.

Gene expression data were analyzed according to the xCell algorithm to calculate 34 immune cell scores in each entorhinal cortex sample, and 14 immune cell scores differed between the two groups (Figure 2A). Among these immune cells, nine were lymphocytes, and the remaining five were myeloid cells. Overall, among the differential immune cell types, except for Th2 cells, which were elevated in patients, most of the lymphocyte scores decreased in ADs, including CD4^+^ Tc, CD4^+^ Te, Th1, natural killer (NK), NK T- and pro-B cells, eosinophils, and regulatory T cells. In contrast, most myeloid cells had elevated scores, except for interstitial dendritic cells (iDCs). They included basophils, mast cells, plasma cells, and macrophages. We also compared age and sex differences in immune cell scores in AD samples and found no significant differences in immune cell scores across age groups (Figure 2B). There were no significant differences in immune cell scores between sexes, except for CD4^+^ Tem (Figure 2C). The results indicated that the immune differences may be independent of sex and age. Using t-distributed stochastic neighbor embedding (tSNE) to reduce the dimension of the data according to the expression of the marker genes in the two groups, patients and controls could be roughly distinguished. (Figure 3A). The ratio of differential genes to cell marker genes is shown in Figure 3B, and the number of csDEGs accounted for less than 10% of the corresponding cell marker genes.

Considering the age difference between the AD and control groups, we used PSM to perform 1:1 matching between the two groups to investigate whether there was an effect of age on immune infiltration. There were 18 samples in each group after matching (Table 2), and there was no difference in age (Figure 3C). We performed immune infiltration analysis using xCell and found that most of the results were consistent with those of prior matching (Figures 3D,E). The results were different for eosinophils, mast cells, and NK cells, in addition to significant differences in gamma delta T cells (*p* < 0.05) and monocytes (*p* < 0.05). Since most of the results did not significantly change after matching for age considering that the reduction in sample size may lead to poorer feasibility of the results, we used the data before matching for further analysis.

**TABLE 2:** Characteristics of the entorhinal cortex tissue in AD and control samples after propensity score matching.

	Controls	ADs	*p*
Age (=<80/> 80)	11/7	11/7	1
Sex (Male/Female)	11/7	8/10	0.317

### Correlation between csDEGs and immune cells

**FIGURE 4:**
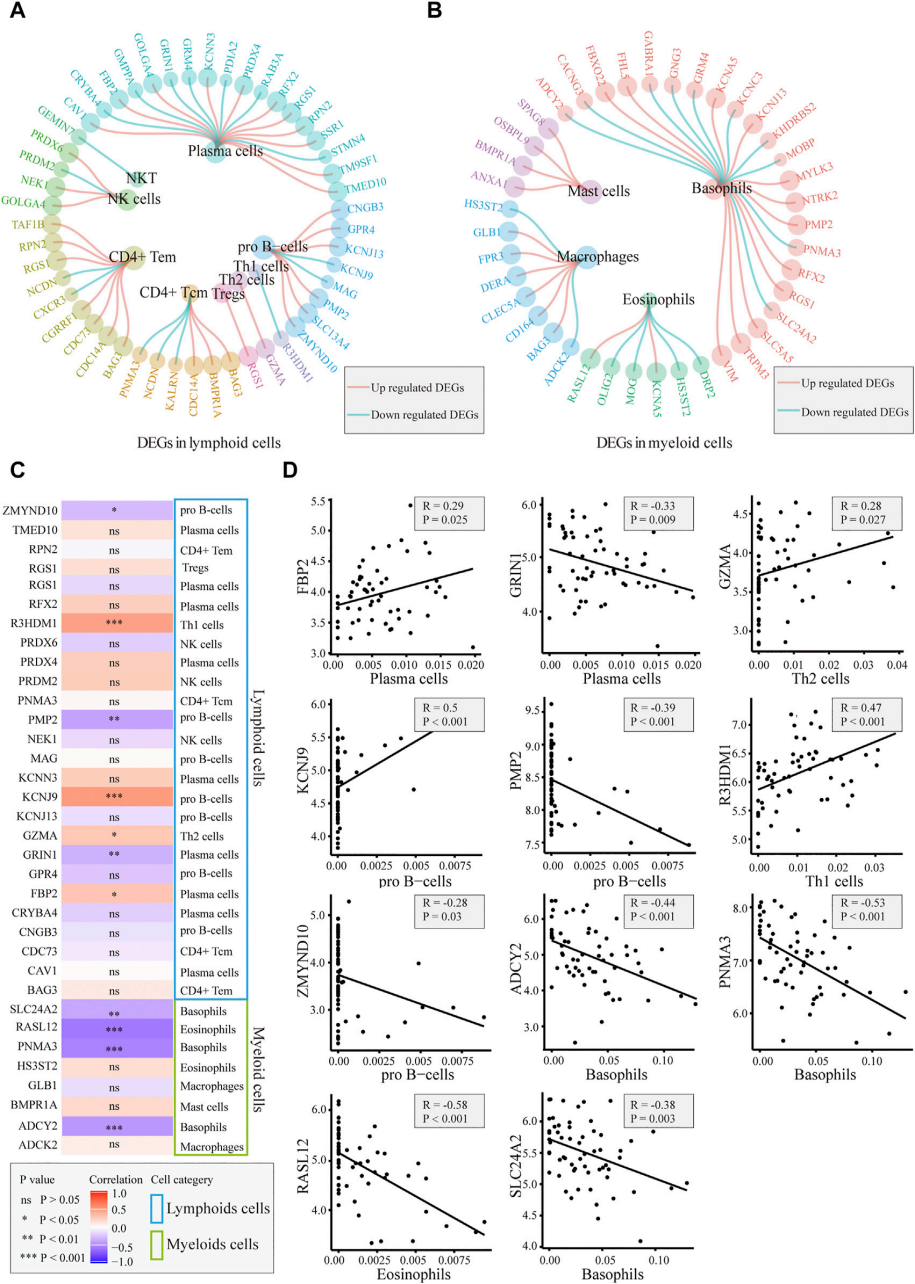
Correlation between immune cells and related gene detection. **(A)**. Differentially expressed marker genes in lymphoid cells. **(B)**. Differentially expressed marker genes in myeloid cells. **(C)**. Heatmap of the correlation between differentially expressed marker genes and corresponding immune cells. **(D)**. Plot of the correlation between differentially expressed marker genes and corresponding immune cells: *FBP2*, *GZMA*, *KCNJ9*, and *R3HDM1* are positively correlated with the corresponding cells. *GRIN1*, *PMP2*, *ZMYND10*, *ADCY2*, *PNMA3*, *RASL12*, and *SLC24A2* were significantly negatively correlated with the corresponding cells.

We explored whether the differences in immune cells were caused by differentially expressed marker genes. The DEGs in the different immune cells are shown in Figures 4A,B. In basophils, mast cells, macrophages, and Th2 cells, most of the differential marker genes were elevated in patients. This finding indicated that the differences in these cells in the AD group may be related to the different marker genes. We performed a correlation analysis between the expression levels of the different marker genes and the corresponding cell scores, and the results showed that *FBP2*, *GZMA*, *KCNJ9*, and *R3HDM1* were positively correlated with these cells, whereas *GRIN1*, *PMP2*, *ZMYND10*, *ADCY2*, *PNMA3*, *RASL12*, and *SLC24A2* were significantly negatively correlated (Figures 4C,D). The results also suggested that these genes may contribute to cellular differences between patients and controls.

### Correlation in immune cells

**FIGURE 5:**
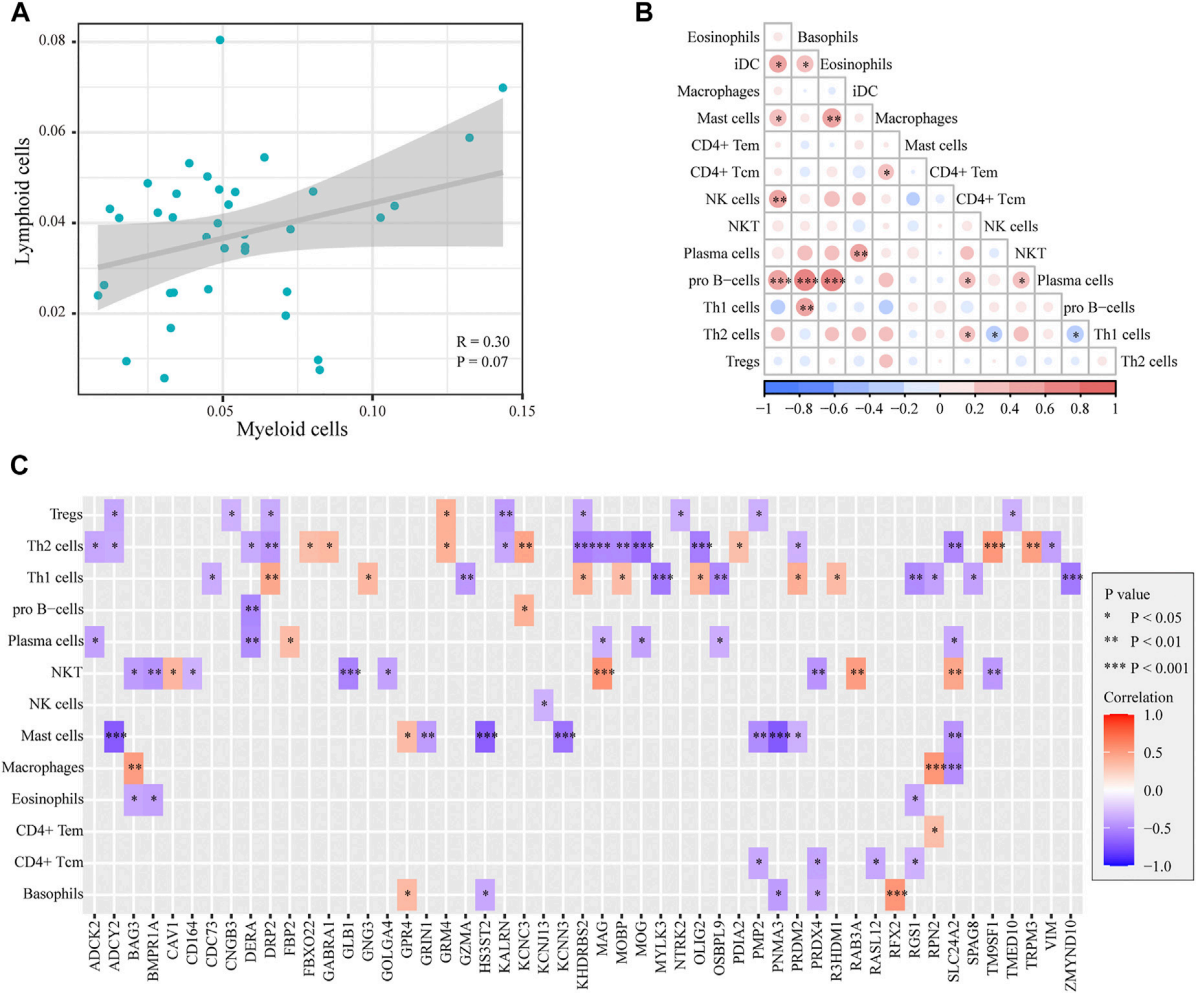
Correlation between myeloid cells and lymphoid cells in ADs. **(A)**. Plot of the correlation between myeloid cells and lymphoid cells: no significant difference was detected (*p* = 0.07). **(B)**. Heatmap of the correlation between immune cells: a close correlation between immune cells. **(C)**. Heatmap of the correlation between cell scores and genes: 51 marker genes were significantly associated with the differentially expressed cells.

To reveal the interrelationships between immune cells in AD samples, we performed the Pearson correlation analysis between intercellular scores in immune cells. The correlation between myeloid cells and lymphoid cells is shown in Figure 5A, and there was no significant correlation between myeloid cells and immune cells (cor = 0.3, *p* = 0.07). The correlation between immune cells is shown in Figure 5B. There was a close association between differential immune cells, most of which were positively correlated, such as basophils and iDCs, mast cells, NK cells, pro-B cells, eosinophils, iDCs, pro-B cells, and Th1 cells. In addition, Th2 cells were negatively correlated with NKT and Th1 cells. We further explored the potential genes leading to cell-to-cell correlation using the correlation analysis between marker genes and cell scores. A total of 51 marker genes were significantly associated with the differentially expressed cells (Figure 5C). Among these genes, 37 immune genes were associated with innate immunity.

### Drug-targeted immune genes associated with innate immunity

**FIGURE 6:**
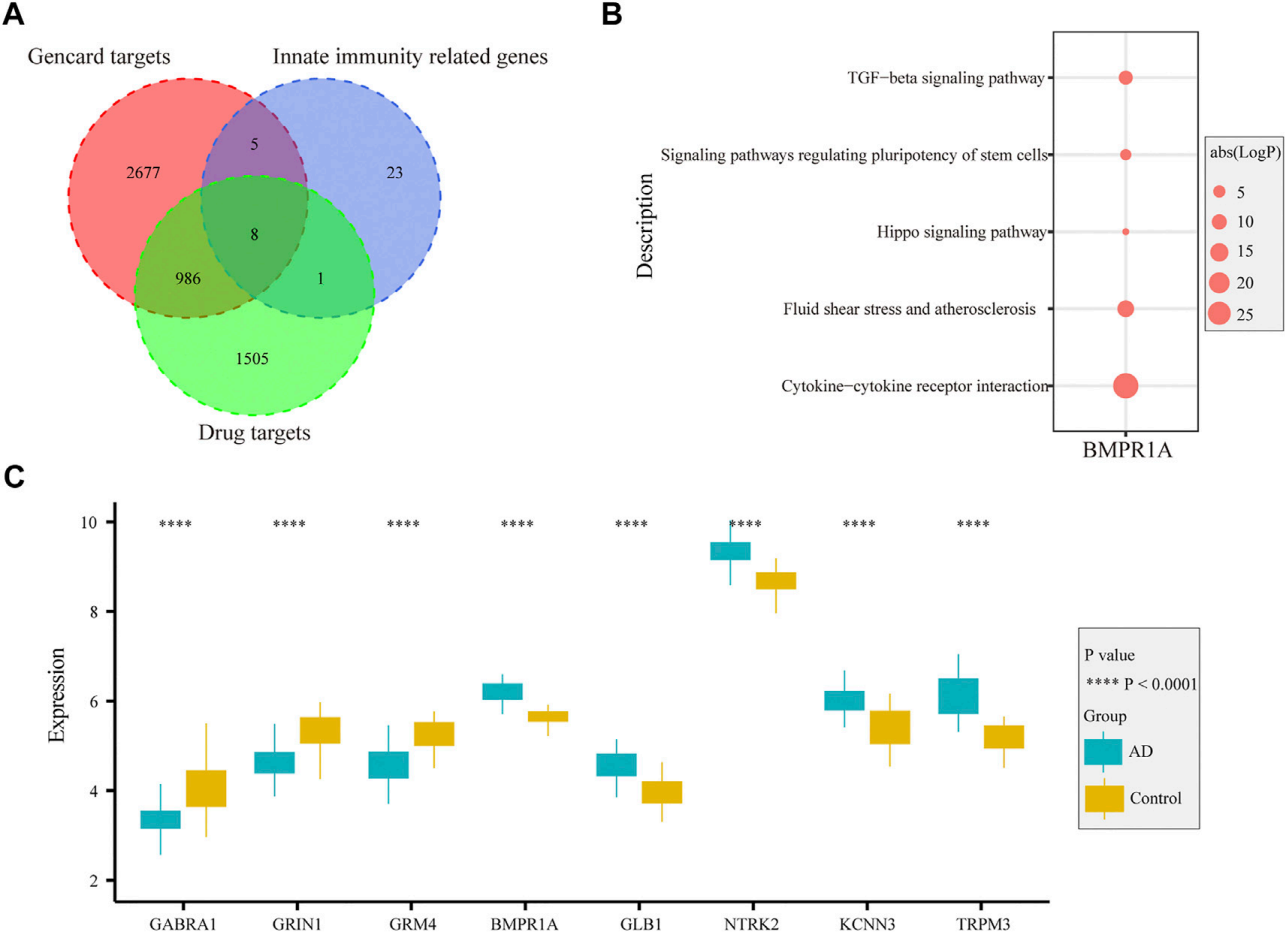
Function and expression of drug-targeted genes associated with myeloid cells in AD. **(A)**. Eight drug-targeted genes associated with myeloid cells for AD were selected. **(B)**. Pathways from KEGG analysis with 81 irDEGs associated with drug-targeted genes. **(C)**. Boxplot displaying the expression of eight drug-targeted genes in the entorhinal cortex between AD and control samples: *GABRA1*, *GRIN1*, and *GRM4* are significantly increased in AD samples, whereas *BMPR1A*, *GLB1*, *NTRK2*, *KCNN3*, and *TRPM3* are decreased in AD samples.

**FIGURE 7:**
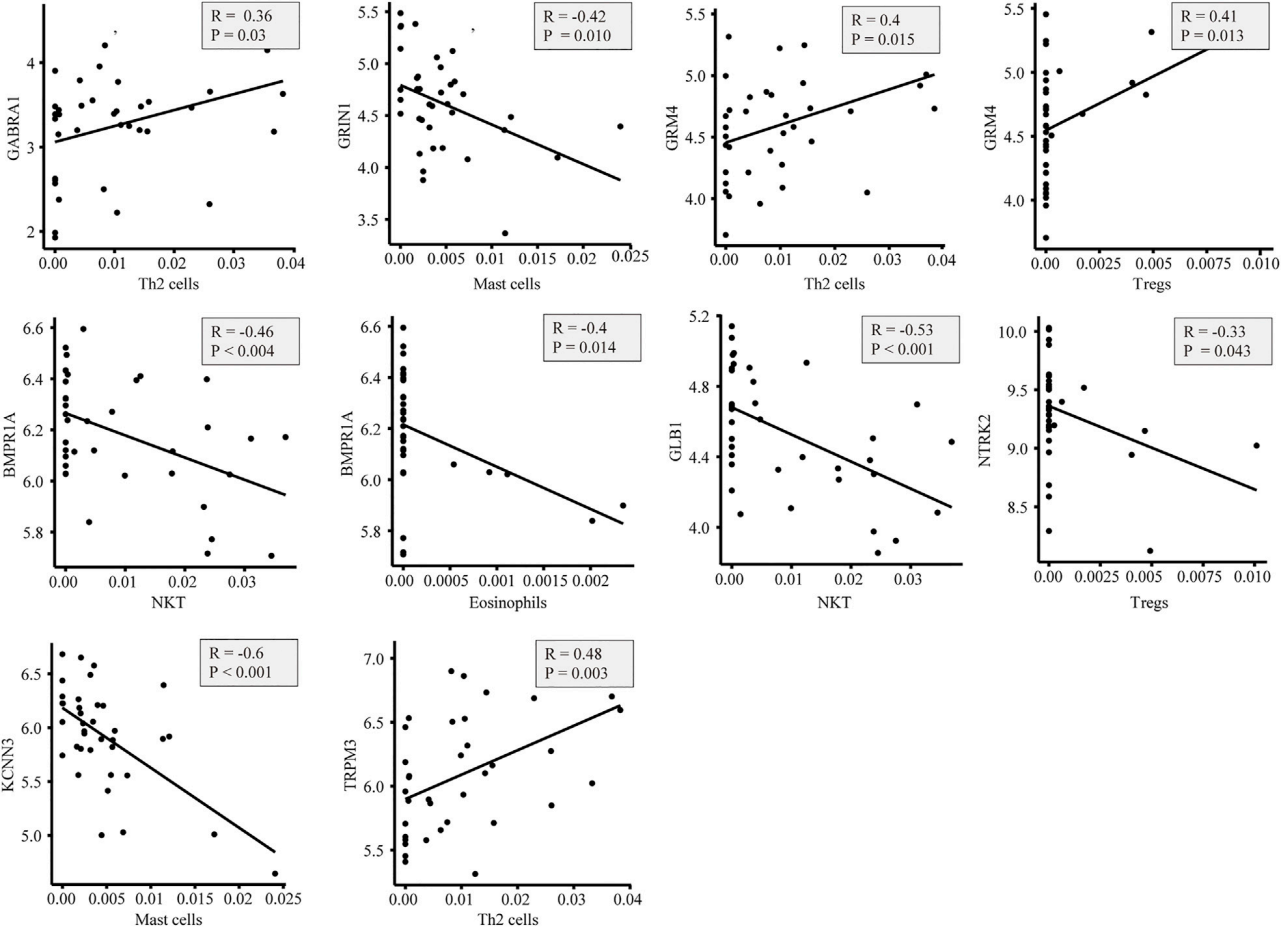
Plot of the correlation between eight genes associated with immune cells: there is a close correlation between genes and immune cells.

**FIGURE 8:**
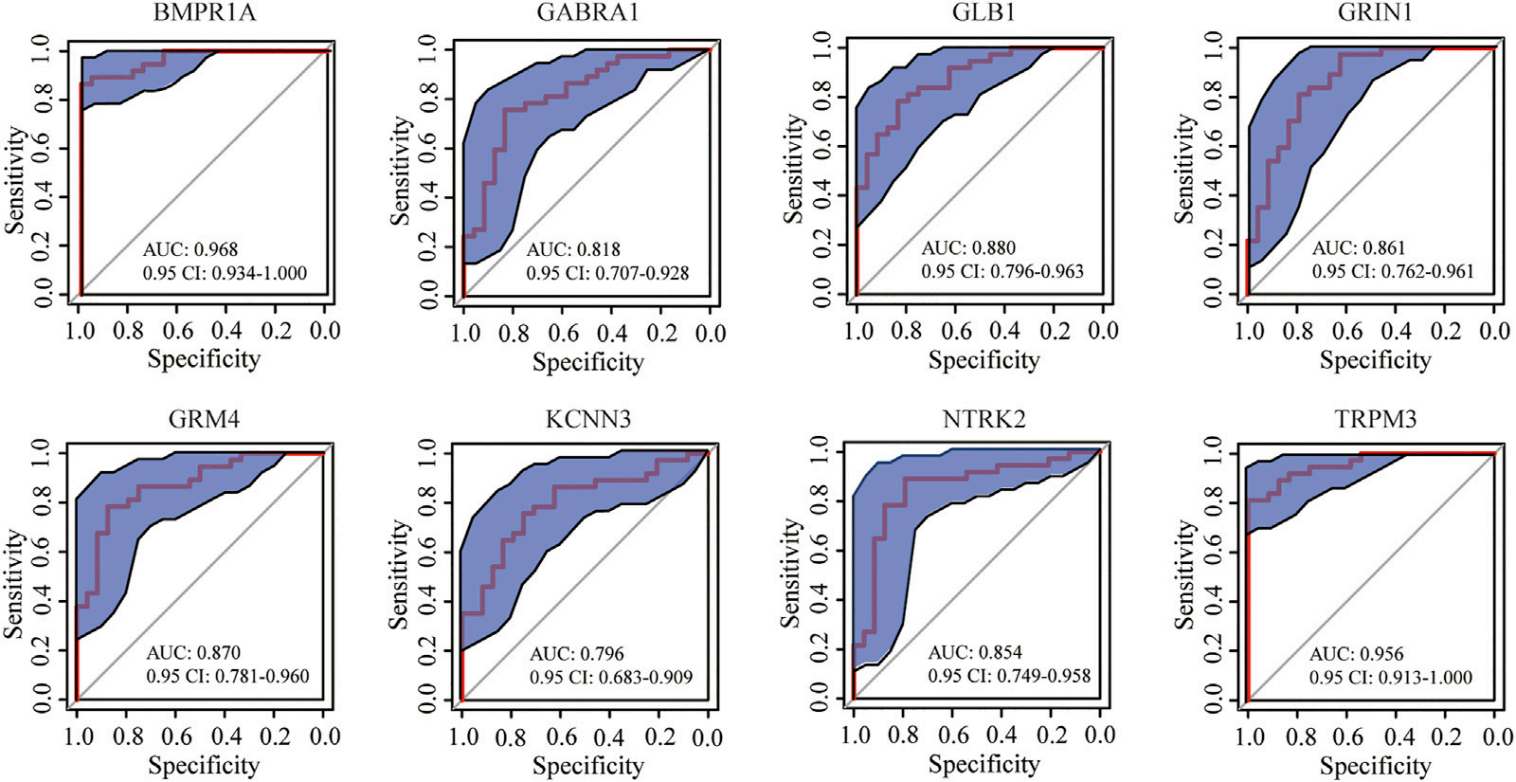
ROC curve of eight genes in predicting AD samples: blue area represents the 0.95 CI value of each gene for the prediction of AD; red curve is the ROC curve of eight genes in predicting AD; almost all AUCs of the gene were higher than 0.8, and the AUCs for *BMPR1A* and *TRPM3* were higher than 0.95.

**FIGURE 9:**
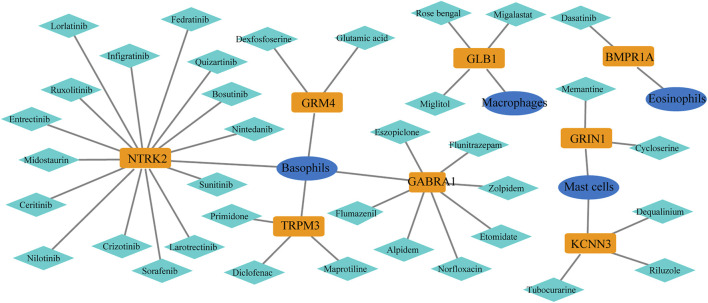
Potential drugs of selected genes from the PharmGKB database: 36 drugs targeted these eight genes.

In the GeneCards database, there are 3,676 genes related to AD according to the inferred functionality scores (>40). Moreover, 2,500 genes were identified as drug targets in the PharmGKB database. Through intersection analysis, we finally identified eight potential therapeutic target genes acting on innate immune cells: *GABRA1*, *GRIN1*, *GRM4*, *BMPR1A*, *GLB1*, *NTRK2*, *KCNN3*, and *TRPM3* (Figure 6A). According to the enrichment analysis results in Metascape, *BMPR1A* participated in cytokine–cytokine receptor interactions, fluid shear stress, and atherosclerosis, Hippo signaling pathway, signaling pathways regulating pluripotency of stem cells, and the tumor growth factor (TGF)-beta signaling pathway (Figure 6B). These pathways may play important roles in the association with immune cells involved. The expression levels of these eight genes in the AD and control groups are shown in Figure 6C. *GABRA1*, *GRIN1*, and *GRM4* were significantly increased in AD, whereas *BMPR1A*, *GLB1*, *NTRK2*, *KCNN3*, and *TRPM3* were significantly decreased (Figure 6C). The significant correlation between the eight genes and immune cells is displayed in Figure 7. We proceeded to test the diagnostic value of the gene expression levels to detect AD in our cohort using the ROC analysis. Almost all AUCs of genes were higher than 0.8, and the AUCs for *BMPR1A* and *TRPM3* were higher than 0.95, confirming that these genes can predict AD with high sensitivity and specificity, despite the small sample size (Figure 8). There were 36 drugs targeting these eight genes, including memantine, cycloserine, riluzole, and diclofenac sodium, which have been reported to be beneficial in reducing the incidence of AD (Figure 9).

### Prediction of candidate miRNAs

**FIGURE 10:**
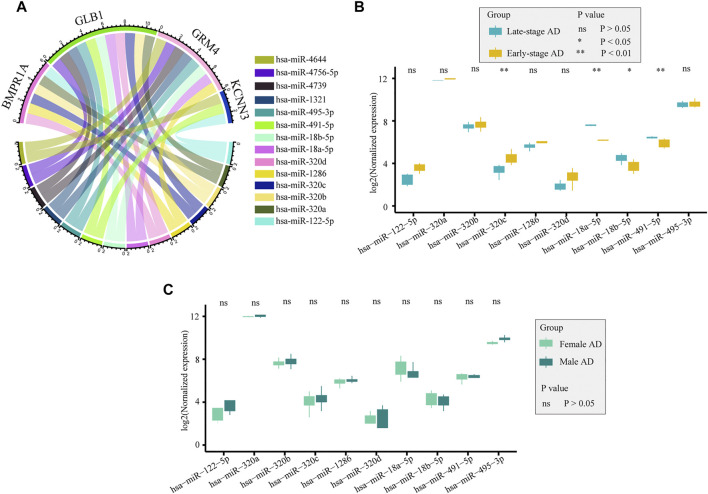
Potential miRNAs regulated selected genes. **(A)**. Potential miRNAs regulating selected genes were obtained from the ENCORI database. **(B)**. Expression of miRNAs between early-stage and late-stage ADs from GSE48552: *hsa-miR-320c*, *hsa-miR-18a-5p*, *hsa-miR-18b-5p*, and *hsa-miR-491-5p* were differentially expressed between the two groups. **(C)**. Expression of miRNAs between females and males ADs from GSE48552: no significant difference was detected between the two groups.

miRNAs play a significant role in immune responses, such as maturation, proliferation, differentiation, and activation. Using the ENCORI database, we predicted miRNAs that may regulate the expression of drug-targeted genes. A total of 14 miRNAs, which may be involved in regulating gene expression and thus affecting immune infiltration, were screened as those with clipExpNum > 2 and that regulated > 2 target immune genes (Figure 10A). They participate in the regulation of immune infiltration by regulating the expression of *BMPR1A*, *GLB1*, *GRM4*, and *KCNN3*. In GSE48552, 10 miRNAs were detected from six early-stage AD patients and six late-stage AD patients, and four miRNAs had different expression levels in different disease states (Figure 10B). *hsa-miR-320c* was highly expressed in early-stage AD, whereas *hsa-miR-18a-5p*, *hsa-miR-18b-5p*, and *hsa-miR-491-5p* were highly expressed in late-stage AD. We also assessed the expression of those miRNAs between sexes in AD, and no significant difference was detected (Figure 10C).

## Discussion

In this study, we aimed to describe the immune cell landscape and related genes that cause specificity in the entorhinal cortex between patients with AD and control patients. We found that 81 irDEGs between patients with AD and controls, such as those involved in chemokine signaling, IL-17 signaling, Th17 cell differentiation, B-cell receptor signaling, hematopoietic cell differentiation, T-cell receptor signaling, C-type lectin receptor signaling, NK cell–mediated cytotoxicity, and Th1 and Th2 cell differentiation, were enriched in several immune system pathways. The immune cell landscape of brain tissues showed that several lymphocyte scores were decreased in AD, including CD4^+^ Tc, CD4^+^ Te, Th1, NK, NKT, pro-B cells, eosinophils, and Tregs. Significantly increased basophils, mast cells, and plasma cells, all of which are myeloid cells, were discovered in AD. The involvement of innate immunity in AD progression has been revealed at the transcriptomic level. In addition, the correlation between marker genes and immune cells detected potential genes that contributed to the immune specificity in the two groups. A close correlation was observed between the differentially scored immune cells. Finally, eight target genes and 36 drugs that may act on innate immunity were identified, which showed a high AUC in the identification of AD and may provide new strategies for AD treatment.

Neuroinflammation has a significant effect on the pathophysiology of AD (Heneka et al., 2015; Ransohoff, 2016; Lindestam Arlehamn et al., 2019). Infiltrating immune cells in border regions can affect the brain by secreting cytokines, modulating adjacent epithelial and ependymal cells, and altering cerebrospinal fluid composition (Cugurra et al., 2021). These cells are involved in tissue homeostasis, and they may enter the brain parenchyma when abnormalities occur. Studies have shown that impaired meningeal lymphatic function may be a factor in the aggravation of AD pathology (Louveau et al., 2016; Da Mesquita et al., 2018). Similarly, dysfunction of the meningeal lymphatic system has been implicated in the pathogenesis of other classic autoimmune neurodegenerative diseases, such as multiple sclerosis and autoimmune encephalitis (Hsu et al., 2019; Schwartz et al., 2019). In AD, neuroinflammation is not a passive system activated by the emergence of senile plaques and neurofibrillary tangles but instead plays an equally (or greater) role in the pathogenesis of plaques and tangles themselves (Zhang et al., 2013). The important role of neuroinflammation is supported by the findings that immune receptor genes, such as *TREM22* and *CD33*, are associated with AD (Bradshaw et al., 2013; Griciuc et al., 2013; Guerreiro et al., 2013). Innate immunity plays a major immune role in AD (Heppner et al., 2015). Innate immune cell hyperexcitability was reported to be associated with cognitive decline (Nam et al., 2022). In a murine amyloidosis model, IFN-I signaling represents a critical module within the neuroinflammatory network of AD and prompts concerted cellular states that are detrimental to memory and cognition (Roy et al., 2022). Collective histological, bioinformatics and molecular analyses highlight the permanent activation of microglia, the brain’s resident immune cells, and the association of many AD risk polymorphisms and rare variants with microglia and innate immunity (Zhang et al., 2013; Huang et al., 2017). Scientists have recognized these and have focused their efforts on therapies aimed at modulating innate immunity. In the present study, we found that the AD group had significantly lower scores for eosinophils, macrophages, NK, NKT, and Treg cells, while higher scores for basophils, mast cells, and macrophages in AD were detected. This result also supports the important role of innate immunity in the development of AD.

In addition to characterizing differences in immune cell profiles between patients with AD and controls, we identified eight genes with potential roles in innate immune cells and 36 drugs with potential therapeutic effects by correlation analysis and combining the GeneCards and PharmGKB databases. These genes and drugs may provide evidence for the treatment of AD. *BMPR1A* encodes a morphogenetic protein receptor. The ligands of these receptors are members of the TGF-β superfamily involved in the regulation of cell proliferation, differentiation, and apoptosis and therefore play essential roles during embryonic development and pattern formation (Morikawa et al., 2016). TGF-β regulates a variety of important cell and tissue functions, such as cell growth and differentiation, angiogenesis, extracellular matrix production, immune function, cell chemotaxis, apoptosis, and hematopoiesis (Flanders et al., 1998). In this analysis, the TGF-β signaling pathway was significantly enriched in KEGG analysis, indicating its critical function in the neuroinflammation of AD. Chemokines are cytokines that orchestrate innate and adaptive immune responses and are differentially regulated in several neuroinflammatory disorders (Charo & Ransohoff, 2006). Our previous analysis provides evidence regarding viral infection in AD development (Sun et al., 2022). In our study, we found that the differential expression of *BMPR1* was significantly related to the occurrence of AD, which may provide new evidence for the treatment of AD in the future. In our analysis, we also found that *NPC2*, *METTL7A*, and *ARL5A* were highly expressed in patients with AD compared to controls. According to previous reports, *NPC2* is considered to be closely related to lipid metabolism (Awan et al., 2022) and tumor metastasis (de Araujo et al., 2021). The relevance of *METTL7A* to lipid metabolism has also received attention (Yi et al., 2020). *APOE*, a key gene in the development of AD, encodes a multifunctional protein with central roles in lipid metabolism (Liu et al., 2013). These studies all indicate that lipid metabolism may play an important role in AD development. *ARL5A* belongs to the ARF family, which are members of the *Ras* gene superfamily of GTP-binding proteins that are involved in a variety of processes, such as cellular communication, endoplasmic reticulum binding, vesicle transport, and protein synthesis (Lin et al., 2002; Wang et al., 2005). The association between *ARL5A* and AD has not been reported and needs to be further studied.

These results suggest that a number of drugs act on innate immune cells through the eight immune genes identified and that they may play an important role in the prevention and treatment of AD. For example, memantine, cycloserine, riluzole, and diclofenac sodium have all been reported to have beneficial effects on reducing the incidence of AD. Memantine, an N-methyl-d-aspartate receptor (NMDAR) antagonist, is clinically quite effective for behavioral symptoms and is often added to cholinesterase inhibitors to enhance their effects, whereas aducanumab has recently been approved for amyloidosis (Langa et al., 2004; Giacobini et al., 2022). Aducanumab is used to mitigate the neurotoxicity associated with AD and other neurodegenerative disorders. Memantine blocks the NMDAR subtype of the glutamate receptor, preventing excessive activation of the glutamate receptor while allowing normal activity (Langa et al., 2004). Its blockade antagonizes the overactive glutaminergic system in the central nervous system (CNS), which is hypothesized to be involved in the neurotoxicity of AD. NMDA encephalitis is an important autoimmune encephalitis, and its associated syndromes and immune-mediated mechanisms have been described (Muñoz-Lopetegi et al., 2020). Our results suggest that memantine may exert a therapeutic effect by affecting *GRIN1* expression in mast cells. These results may offer new perspectives for the treatment of NMDA autoimmune encephalitis and AD with memantine. d-cycloserine exhibits partial agonist activity at the glycine site of the NMDA subtype of the glutamate receptor, promoting receptor activation and improving cognition and memory, which has been validated as a cognitive benefit in patients with AD (Bowen et al., 1992; Tsai et al., 1999). Riluzole, the glutamate modulator, is FDA-approved for the treatment of amyotrophic lateral sclerosis, with potential benefits for cognition, aging, and structural and molecular markers of AD (Matthews et al., 2021). Diclofenac is chemically related to the finasteride class of NSAIDs and has been shown to improve cognition in two independent studies using mouse models of AD (Joo et al., 2006; Daniels et al., 2016). It is also associated with a reduced risk of developing AD (Stuve et al., 2020).

miRNAs play important gene regulatory roles in animals and plants by pairing with mRNAs of protein-coding genes to direct their posttranscriptional repression (Bartel, 2009). Therefore, we also predicted miRNAs that might regulate the expression of drug-targeted genes. We found that these 14 microRNAs might be involved in regulating gene expression, thereby affecting immune infiltration. *hsa-miR-320a*, *hsa-miR-495*, and *hsa-miR-122-5p* have been reported to be associated with autoimmune disease-related outcomes (Yao et al., 2019; Cordes et al., 2020; Ni & Leng, 2020; Fu et al., 2021). It is noteworthy that the reports for *hsa-miR-320a*, *hsa-miR-320b*, and *hsa-miR-320c* involved central immunity (Regev et al., 2018). Our results predict the important role of autoimmunity in AD development in the related assessment of miRNAs, and they reveal powerful new endogenous combinatorial therapeutic targets.

In conclusion, this study attempted to clarify the possible mechanism of the immune microenvironment involved in the occurrence and development of AD by analyzing the immune microenvironment of the entorhinal cortex of patients with AD and to describe the association between genes on drugs and immune cells. However, this study still has certain limitations. First, the application of drugs affects transcriptome expression, but we were not able to obtain reliable information on drug usage from GEO dataset. Second, age may have an effect on immune infiltration, but considering that the smaller sample size would lead to less reliable results, our study did not use age-matched data for analysis. Third, the results were not validated with biological experiments, which are strictly limited by ethics. In the future, we will further study the role of the immune microenvironment in the pathogenesis of AD using more samples and animal models. In conclusion, our study describes the specificity of the immune cell landscape and associated genes contributing to AD in the entorhinal cortex, which provides new insights into the treatment of AD.

## Data Availability

The datasets presented in this study can be found in online repositories. The names of the repository/repositories and accession number(s) can be found below: https://www.ncbi.nlm.nih.gov/geo/, GSE118553, GSE48552.
