# Supplementary figures and images for: Landscape of immune infiltration in entorhinal cortex of patients with Alzheimerʼs disease
Source: Front Pharmacol. 2022 Sep 28;13:941656. doi: 10.3389/fphar.2022.941656 (PMC9557331; doi:10.3389/fphar.2022.941656)

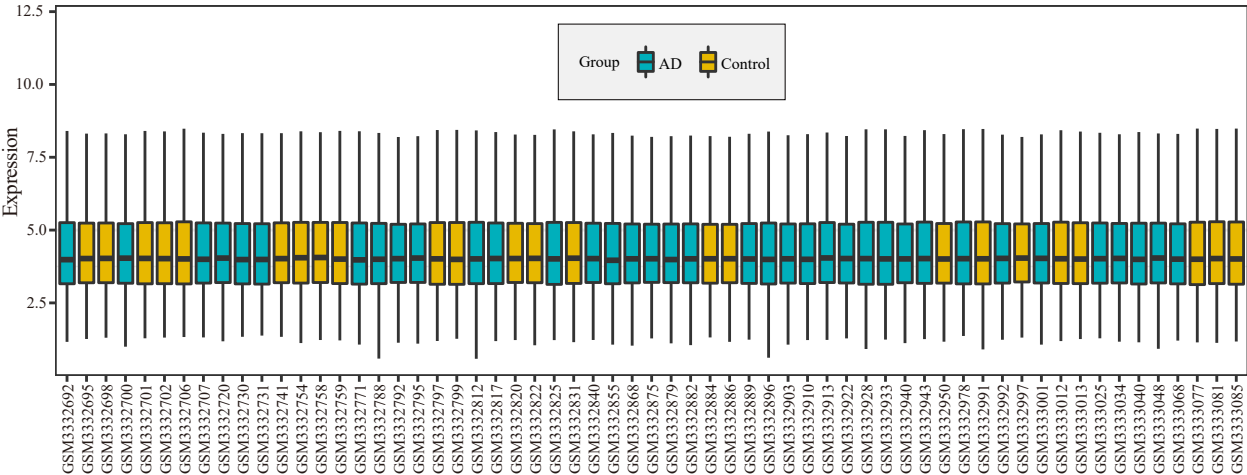

Supplement: Supplementary file 1 [file DataSheet1.PDF]
